# Bacterial Community Structure and Environmental Driving Factors in the Surface Sediments of Six Mangrove Sites from Guangxi, China

**DOI:** 10.3390/microorganisms12122607

**Published:** 2024-12-17

**Authors:** Ying Liu, Songze Chen, Jinyu Liang, Jingjing Song, Yue Sun, Riquan Liao, Mingzhong Liang, Hongming Cao, Xiuhao Chen, Yuxia Wu, Liting Bei, Yuting Pan, Baishu Yan, Yunru Li, Yun Tao, Rongping Bu, Bin Gong

**Affiliations:** 1 Pinglu Canal and Beibu Gulf Coastal Ecosystem Observation and Research Station of Guangxi, Guangxi Key Laboratory of Beibu Gulf Marine Biodiversity Conservation, College of Marine Sciences, Beibu Gulf University, Qinzhou 535011, China; liuying06shengke@163.com (Y.L.); 13099879245@163.com (J.L.); songjingjing@bbgu.edu.cn (J.S.); sunyue7134@163.com (Y.S.); liaorq@bbgu.edu.cn (R.L.); bhlmz@163.com (M.L.); redbull88@163.com (H.C.); 18176330218@163.com (X.C.); 19977490424@163.com (Y.W.); m18077017489@163.com (L.B.); pyt190602@163.com (Y.P.); ybsqwer123@163.com (B.Y.); liyunru0325@163.com (Y.L.); 19877624429@163.com (Y.T.); 2 Shenzhen Ecological and Environmental Monitoring Center of Guangdong Province, Shenzhen 518049, China; songzechen2012@sina.com

**Keywords:** mangrove wetlands, bacterial communities, environmental driving factors, spatial distribution, 16S rRNA sequencing

## Abstract

Mangroves, as blue carbon reservoirs, provide a unique habitat for supporting a variety of microorganisms. Among these, bacteria play crucial roles in the biogeochemical processes of mangrove sediments. However, little is known about their community composition, spatial distribution patterns, and environmental driving factors, particularly across the large geographical scales of mangrove wetlands. In this study, the composition and spatial distribution of the bacterial community structure and its response to fifteen physicochemical parameters (including temperature, pH, salinity, moisture, clay, silt, sand, organic carbon (OC), total nitrogen (TN), total phosphorus (TP), inorganic phosphorus (IP), organic phosphorus (OP), δ^13^C, δ^15^N, and carbon/nitrogen ratio (C/N ratio)) were characterized in 32 sampling locations of six different mangrove habitats from Guangxi, China, applying 16S rRNA gene high-throughput sequencing technology and correlation analysis. Our results indicated that the spatial distribution patterns in bacterial communities were significantly different among the six different mangrove sites, as evidenced by NMDS (non-metric multidimensional scaling), ANOSIM (analysis of similarity), and LDA (linear discriminant analysis) analysis. Composition analysis of bacterial communities showed that overall, Chloroflexi (8.3–31.6%), Proteobacteria (13.6–30.1%), Bacteroidota (5.0–24.6%), and Desulfobacterota (3.8–24.0%) were the most abundant bacterial phyla in the mangrove surface sediments. Redundancy analysis (RDA) further highlighted that salinity, δ^13^C, temperature, δ^15^N, and silt were the most critical environmental variables influencing the composition of bacterial communities across the whole mangrove samples. Notably, Chloroflexi, one of the most abundant bacterial phyla in the mangrove wetlands, displayed a significantly positive correlation with OC and a negative correlation with δ^13^C, suggesting its essential role in the degradation of terrestrial-derived organic carbon. These findings support the current understanding of the roles of the bacterial communities and their interactions with environmental factors in diverse mangrove ecosystems.

## 1. Introduction

Mangroves, distributed all over the world, are diverse, complex, and productive ecosystems composed of woody plants in tropical and subtropical estuaries and shorelines [1]. Mangroves are characterized by a unique acidic, organic-rich, and hypoxic-reducing sedimentary environment [2]. Mangrove ecosystems play important roles in carbon fixation, shoreline stability, erosion mitigation, and water purification [1,3,4]. They possess various biological resources and thus support a rich diversity of living organisms [4]. 

Bacterial communities are important components of the mangrove ecosystem and they play essential roles in facilitating the biogeochemical cycling of C, N, P, S, and Fe in mangrove sediments and promote the growth of mangrove plants by generating phytohormone and siderophore [3,5,6,7,8]. Most importantly, the degradation and turnover of a large amount of organic matter, which originated from the withering of mangrove branches and leaves, is mediated largely by bacterial communities in the mangrove sediments [9,10]. For instance, Chloroflexi have a strong capability to degrade recalcitrant OC such as lignin in mangrove sediments [10,11,12]. As sulfate reduction bacteria, Desulfobacterota tend to stimulate the metabolic activities in transformations of S, Fe, and N compounds in subtropical mangrove sediments [11,13,14]. Therefore, the maintenance and restoration of microbial communities are fundamental to the productivity, survival, conservation, and recovery of mangroves in intertidal regions [4]. However, previous studies only focus on sediment bacterial communities among several distinct sites of mangrove forest [15,16]. Little is known about the large-scale biogeographic distribution patterns of bacterial communities in mangrove ecosystems.

Acting as a transitional zone linking terrestrial and marine environments, mangroves are affected by variable physicochemical conditions, including temperature, salinity, pH, grain size, sediment depth [17], and sediment physicochemical properties [10,16,18,19,20]. Changes to a variety of environmental factors in the sedimentary environment of mangroves could directly or indirectly influence the diversity, composition, and metabolic activities of bacterial communities [1,3,16,21]. For instance, Bai et al. (2013) indicated that the microbial community structure are largely shaped by surrounding environmental variables, e.g., salinity, TN (total nitrogen), TC (total carbon), pH, and C/N (carbon/nitrogen) ratio in Zhangjiang Estuary Mangrove National Natural Reserve [5]. Also, the composition of microbial communities is regulated by altering redox conditions and organic carbon levels in the mangrove sediments [22]. Moreover, the biogeographic distribution of bacterial communities in mangroves across China is driven by the wetland trophic status and salinity [6]. It is well known that different mangrove habitats have varied environmental factors, and specific bacterial community structures have formed to adapt to the complicated mangrove wetlands [16,23]. However, the key environmental factors driving the biogeographic distribution of the mangrove bacterial communities are still poorly understood.

Six representative mangrove wetlands in Guangxi, China (Fangcheng Port Mangrove Natural Reserve, FCG; Maowei Sea Mangrove Natural Reserve, MWH; Shajing Port mangrove wetland, SJG; Xiandao Park mangrove wetland, XD; Sanniang Bay mangrove wetland, SNW; Bei Sea Mangrove Natural Reserve, BH) were selected in this study. FCG is located in Fangcheng Port (Guangxi, China). MWH is located in Qinzhou (Guangxi, China), and the average annual air temperature and rainfall are 22.4 °C and 2150 mm, respectively. The seawater of MWH has a mean temperature of 23.5 °C, salinity of 20–23‰, and pH of 7.6–7.8. The mangroves of MWH are typically 1–4 m high and contain various species, such as *Aegiceras corniculatum*, *Avicennia marina*, *Kandelia candel*, and *Rhizophora stylosa* [24]. SJG, XD, and SNW are located in Qinzhou. BH is located in the Bei Sea (Guangxi, China).

The objective of the present study was to investigate the response of the bacterial communities to fifteen physicochemical parameters, including temperature, pH, salinity, moisture, clay, silt, sand, OC (organic carbon), TN (total nitrogen), TP (total phosphorus), IP (inorganic phosphorus), OP (organic phosphorus), δ^13^C, δ^15^N, and C/N ratio, in the surface sediments of six mangrove sites from Guangxi province, China. First, the values of the fifteen physicochemical parameters were measured in 32 surface sediment samples from six different sites of mangrove wetlands. Then, the spatial distribution patterns of the bacterial communities were determined from the surface sediments of the six different mangrove habitats via 16S rRNA gene high-throughput sequencing. Next, the responses of the bacterial communities to various environmental factors in the mangrove sediments were evaluated through redundancy analysis (RDA) and Mantel analysis. The results of this study will be conducive to understanding the roles of the bacterial community and its linkages with environmental factors in the large, geographic-scale mangrove ecosystems.

## 2. Materials and Methods

### 2.1. Sediments Sampling

Surface sediment samples were collected from six representative mangrove wetlands, including Fangcheng Port Mangrove Natural Reserve (FCG), Maowei Sea Mangrove Natural Reserve (MWH), Shajing Port mangrove wetland (SJG), Xiandao Park mangrove wetland (XD), Sanniang Bay mangrove wetland (SNW), and Bei Sea Mangrove Natural Reserve (BH), in August to September 2023 Figure 1. The geographical locations of the six representative mangrove wetlands were different. The latitudes and longitudes of the sites are listed in Table 1. All samples were transferred on ice to the laboratory in four hours. Sediment samples were separated into two sets. One sample set was stored at −80 °C before DNA extraction, and the other set was preserved at −20 °C before physicochemical analysis.

### 2.2. Environmental Parameter Analysis

Temperature, salinity, and pH were measured using a soil multiparameter recorder with a soil-integrated sensor. The samples stored at −20 °C were weighed and recorded as w1. Then, they were weighed again, and recorded as w2 after freeze-dying at −40 °C for 96 h in a freeze dryer (YB-FD-1, Shanghai Yibei Industrial Co., Ltd., Shanghai, China). The moisture content of the sediment sample was equal to [(w1 − w2)/w2] × 100%. After pre-treatment, sediment particle size was measured using a laser particle size analyzer (Mastersizer 3000; Malvern Instruments Ltd., Worcestershire, UK) capable of analyzing sediment grains ranging from 0 to 2000 μm. 

After being treated with 1 M HCl for 24 h to remove inorganic carbon, the OC and TN in sediment samples were detected using a CHNOS Elemental Analyzer (Vario EL-II; Elementar Analysensyteme GmbH, Hanau, Germany). The δ^13^C and δ^15^N isotopic values were determined using the isotope ratio mass spectrometer (Delta XL Plus, Thermo Fisher Scientific, Bremen, Germany).

The abundance of total phosphorus (TP) in surface sediment samples was analyzed using the spectrophotometric phosphomolybdate blue method following a procedure involving the extraction of 0.15 g samples with 1 M HCl after ashing at 550 for 3 h. Similarly, calculation of the abundance of inorganic phosphorus (IP) was carried out involving the extraction of 0.15 g samples with 1 M HCl. Sediment samples were measured alongside the standard coastal marine sediment for the Chinese seas (GBW07314). The organic phosphorus (OP) was equal to TP value minus IP value.

### 2.3. DNA Extraction

The total DNA was extracted from 0.5 g of sediment samples using the FastDNA Spin Kit for Soil (MP Biomedical, Solon, OH, USA) following the manufacturer’s instructions. The obtained total DNA was dissolved in 100 μL DNase-free ddH_2_O. The quantity and quality of the extracted DNA were measured by NANODROP 2000 (Thermo Scientific, Wilmington, DE, USA), and preserved at −80 °C until further analysis.

### 2.4. PCR Amplification of 16S rRNA Gene

The DNA of sediment samples was amplified using the barcoded primers 515F (5′-GTGYCAGCMGCCGCGGTAA-3′) and 926R (5′-CCGYCAATTYMTTTRAGTTT-3′), targeting the V4–V5 region of the 16S rRNA gene [25]. The 50 μL PCR reactions contained 25 μL Premix Taq^TM^ (Takara Biotechnology, Dalian, China), 2.5 μL of each primer (10 μM), 10 ng of DNA, and 19 μL DNase-free ddH_2_O. The PCR amplification was carried out on a thermocycler PCR system (GeneExplorer, BIOER Technology, Hangzhou, China) with the following program: 37 °C for 5 min; 95 °C for 10 min; 25 cycles of 95 °C for 45 s, 50 °C for 45 s, and 68 °C for 90 s; 68 °C for 5 min. Negative and positive controls were contained in each run. All PCR amplicons were visualized on 1% agarose gels and purified using EZNA Cycle Pure Kit (Omega Bio-Tek, Inc., Norcross, GA, USA), then quantified using Qubit 3.0 Fluorometer (Invitrogen, Carlsbad, CA, USA). 

### 2.5. High-Throughput Amplicon Sequencing and Data Processing

The amplicon samples were pooled in equimolar and sequenced on the MGISEQ-2000RS in PE300 mode. The raw sequences were trimmed with cutadapt (sequences with average quality scores lower than 20 were filtered, and primer sequences were cut off) [26]. The trimmed sequences were truncated, denoised, and filtered for chimeras using DADA2 commands. The amplicon sequence variants (ASVs) of 16S rRNA sequences were then classified with the classify-sklearn plugin against the SILVA 138.1 database in QIIME2 [27]. Finally, the mitochondria and unclassified ASVs were removed. These processes produced 44,625–246,852 sequences of 32 different samples in the ASV table. The sequence numbers were normalized to an equal number (44,625) by subsampling in QIIME2 for a later statistical and ecological analysis. The alpha diversity (Shannon, Simpson, ACE, and Chao) of each sample was calculated using Mothur-1.35.1 [28].

### 2.6. Statistical Analyses

Non-metric multidimensional scaling (NMDS) and the analysis of similarity (ANOSIM) were performed using the PRIMER v7 package [29]. Linear discriminant analysis (LDA, https://www.omicstudio.cn/tool, accessed on 18 August 2024) was used to identify potential biomarkers at different taxonomy levels by applying an LDA score threshold of 4.0, and the Kruskal–Wallis significance threshold of <0.001 and <0.05 for bacteria. Redundancy analysis (RDA) was conducted to evaluate the relationships between the bacterial community and the physicochemical variables based on CANOCO 5 software. The Mantel test was performed using Spearman’s correlation based on the R package. The correlation heatmaps between the bacterial community and different environmental factors were evaluated using Spearman’s correlation based on the R package. The phylogenetic tree was constructed using the MEGA v7.0 software [30], and the bubble chart was produced using TBtools software v2.096 [31]. 

## 3. Results

### 3.1. Physicochemical Parameters in Sediments and Sediment Textures

The temperature of the six mangrove surface sediments from Guangxi Province changed from 24.2 to 30.7 °C (average, 27.5 ± 1.7 °C), the highest and lowest values were found at sites MWH-D and BH-C, respectively (Table 1). The pH for the whole sediment samples ranged from 5.8 to 9.0 (7.2 ± 0.7), with the highest and lowest values at sites BH-D and MWH-C, respectively. The salinity for the whole sample ranged from 0.2 to 7.7 psu (3.3 ± 2.1 psu), with the highest and lowest values at sites SNW-E and MWH-A, respectively. The moisture for the whole sample ranged from 19.0 to 67.3% (39.0 ± 12.7%), with the highest and lowest values at sites SNW-E and BH-A, respectively (Table 1).

In general, the textures of the surface sediments in the investigated mangrove sites were mainly clayey silt and sandy silt (Table 1). In the overall sample set, silt (4–64 μm) was the predominant sediment grain type, accounting for 40.6–77.5% (61.7 ± 9.2%), followed by clay (0–4 μm) ranging from 10.2 to 50.0% (20.8 ± 9.5%), while sand (64–2000 μm) was the least dominant grain type changed from 0.4–41.1% (17.5 ± 12.5%) of the total sediment texture types. 

### 3.2. OC, TN, TP, IP, OP, δ^13^C-org, δ^15^N, and C/N Ratio

The percentage concentration of the organic carbon (OC) and the total nitrogen (TN) in the whole sample set ranged from 0.1 to 5.3% (average: 1.7 ± 1.2%) and 0.01 to 0.30% (average: 0.12 ± 0.07%), respectively, with the highest and lowest values occurring at sites SJG-2 and SNW-C, respectively. Briefly, OC was in the ranges 0.1–1.7%, 0.6–2.7% 0.4–3.1%, 1.0–3.1%, 0.2–3.7%, and 0.5–5.3% in sites SNW, BH, FCG, XD, MWH, and SJG, respectively. Similarly, TN was in the ranges 0.01–0.14%, 0.06–0.17%, 0.05–0.21%, 0.03–0.22%, 0.01–0.23%, and 0.04–0.30% in stations SNW, XD, BH, FCG, MWH, and SJG, respectively (Table 1).

The concentration of TP ranged from 3.8 to 11.8, 3.9 to 14.8, 5.4 to 19.4, 13.4 to 21.2, 1.9 to 24.0, and 12.0 to 33.3 μmol/g in sites SNW, BH, FCG, XD, MWH, and SJG, respectively, with the highest and lowest concentrations observed at sites SJG-E and MWH-E, respectively (Table 1). Similarly, the concentration of IP ranged from 2.6 to 8.4, 4.2 to 11.6, 8.9 to 13.2, 0.2 to 19.7, 1.7 to 20.3, and 8.9 to 26.7 μmol/g in sites SNW, FCG, XD, MWH, BH, and SJG, respectively, with the highest and lowest concentrations also occurring at sites SJG-E and MWH-E, respectively. In addition, the concentration of OP ranged from 1.2 to 4.2, 1.2 to 7.9, 4.0 to 7.9, 2.9 to 8.3, 1.7 to 8.7, and 1.7 to 9.9 μmol/g in sites SNW, FCG, XD, SJG, MWH, and BH, respectively, with the highest and lowest concentrations found at sites BH-C and FCG-A, respectively. 

On the other hand, the δ^13^C-org values ranged from −29.5‰ to −21.9‰ (average, −26.0‰ ± 1.8) in the mangrove sampling sites, with the highest and lowest values observed at sites BH-A and SJG-D, respectively. The δ^15^N values in the whole samples ranged from 0.8–8.7‰ (average, 4.8 ± 1.7‰), with the highest and lowest values found at stations MWH-A and XD-E, respectively. The C/N ratios of all sediment samples ranged from 11.5 to 22.6 (average: 16.4 ± 3.0), with the highest and lowest values observed at sites XD-A and BH-C, respectively (Table 1).

### 3.3. Bacterial Diversity and Community Structure in Mangrove Sediments

After quality filtering and chimera removal, a total of 4,131,959 high-quality bacterial sequence reads were obtained from the 32 mangrove sediment samples, with an average of 129,124 bacterial sequence reads per sample. Thus, a total of 28,748 bacterial amplicon sequence variants (ASVs) were identified. 

**Figure 2:**
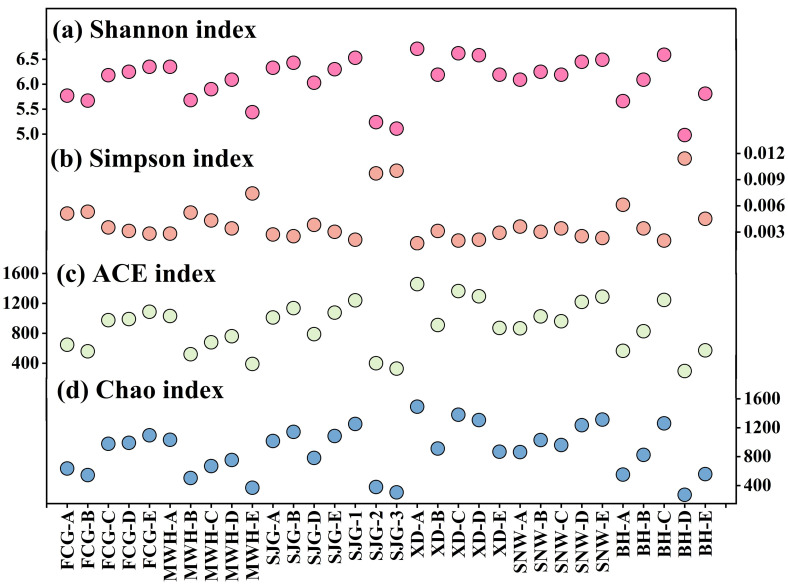
The alpha diversity indices of bacterial communities in the surface sediments of six mangrove sites from Guangxi, China. (**a**) Shannon index; (**b**) Simpson index; (**c**) ACE index; (**d**) Chao index.

The Shannon diversity indexes of bacteria ranged from 5.0 to 6.7 (average: 6.1 ± 0.4), with the highest and lowest values occurred at sites XD-A and BH-D, respectively, in the surface sediments of six mangroves from Guangxi province (Figure 2a). In contrast, the Simpson diversity indexes of bacteria varied from 0.0018 to 0.0115 (0.0042 ± 0.0024), with the highest and lowest values observed at stations BH-D and XD-A, respectively (Figure 2b). On the other hand, the ACE richness indexes of bacteria ranged from 308 to 1469 (899 ± 320), with the highest and lowest values occurring at sites XD-A and BH-D, respectively (Figure 2c). Similarly, the Chao richness indexes of bacteria ranged from 308 to 1484 (901 ± 322), with the highest and lowest values observed at sites XD-A and BH-D, respectively (Figure 2d).

**Figure 3:**
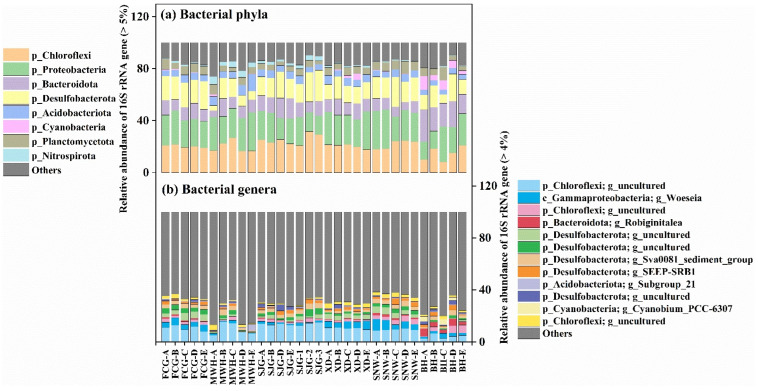
The composition of the bacterial communities at the phylum level (**a**) and at the genus level (**b**), respectively, in the surface sediments of six mangrove stations from Guangxi, China.

At the phylum level, the mangrove bacterial communities were dominated by Chloroflexi (8.3–31.6%), Proteobacteria (13.6–30.1%), Bacteroidota (5.0–24.6%), and Desulfobacterota (3.8–24.0%) in the six representative mangrove surface sediments (FCG, XD, MWH, SJG, SNW, and BH) from Guangxi province (Figure 3a). At the genus level, the dominant bacterial genera mainly included Chloroflexi uncultured genera, *Woeseia* (0.3–8.6%), *Robiginitalea* (0.0–5.9%), *Sva0081_sediment_group* (0.7–4.9%), and *SEEP-SRB1* (0.0–4.9%) in the surface sediments of six mangrove sites (Figure 3b).

### 3.4. The nMDS (Non-Metric Multidimensional Scaling), ANOSIM (Analysis of Similarity), and LDA Analysis (Linear Discriminant Analysis)

**Figure 4:**
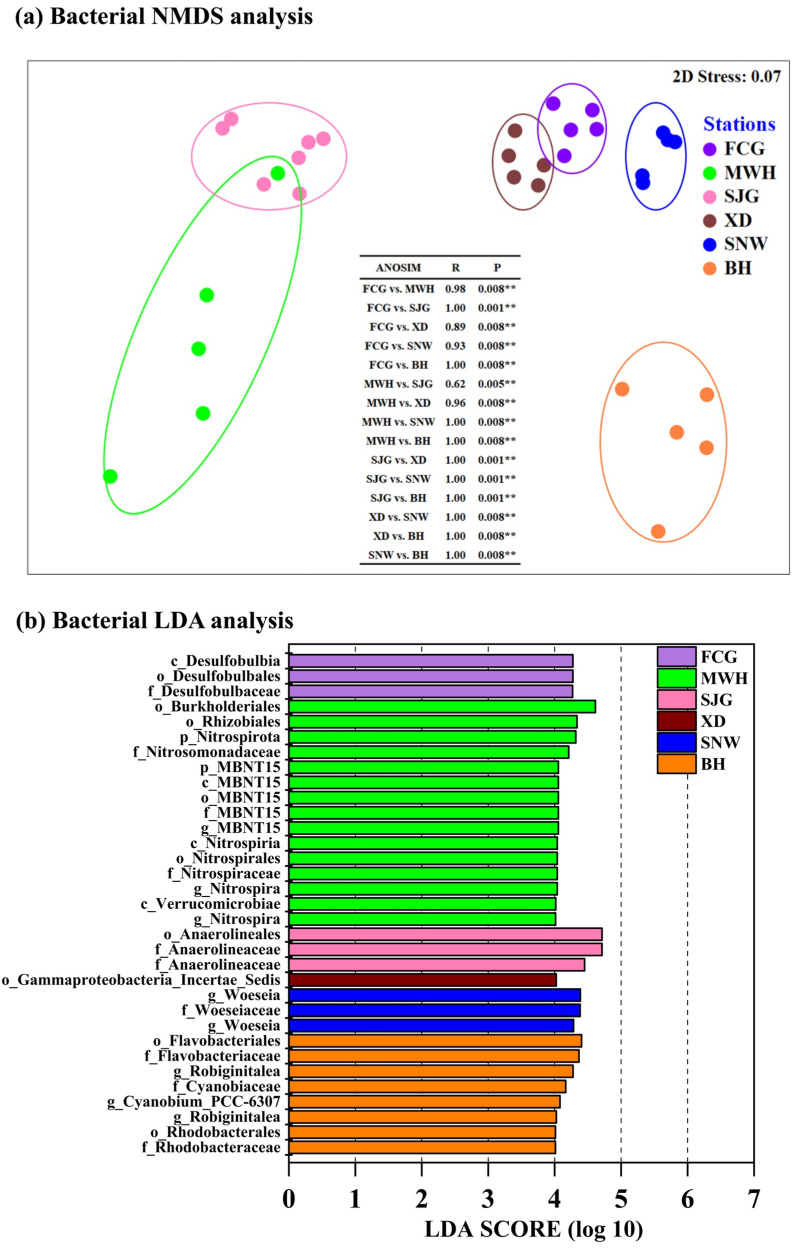
(**a**) Bacterial NMDS (non-metric multidimensional scaling) analysis based on the Bray–Curtis distance metrics and ANOSIM (analysis of similarities) and (**b**) bacterial LDA (linear discriminant analysis) of the surface sediments of six mangrove stations from Guangxi, China. ** indicates *p* < 0.01 in the (**a**).

nMDS and ANOSIM were performed in order to evaluate the similarities in bacterial communities according to different mangrove stations. NMDS analysis showed that a total of six different bacterial clusters were formed based on each site, and ANOSIM also demonstrated that there were significant differences in bacterial community composition between any two mangrove sites (*p* < 0.05) (Figure 4a).

LDA was used to examine the significantly different biomarkers in the bacterial genera among six different mangrove sites. Bacterial LDA results showed that a total of 3, 15, 3, 1, 3, and 8 indicative bacterial taxa were identified at mangrove sites FCG, MWH, SJG, XD, SNW, and BH, respectively. For example, Desulfobulbia, Burkholderiales, and Anaerolineales were significantly enriched at mangrove sites FCG, MWH, and SJG, respectively, whereas members of Gammaproteobacteria_Incertae_Sedis, *Woeseia*, and Flavobacteriales appeared to be significantly enriched at sites XD, SNW, and BH, respectively (*p* < 0.001) (Figure 4b). 

### 3.5. Correlation Between Bacterial Community Composition and Environmental Variables

**Figure 5:**
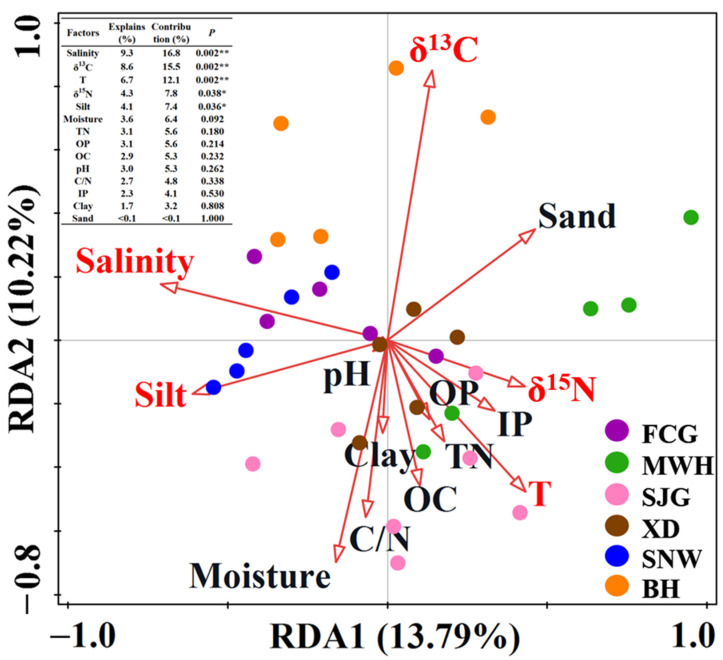
Redundancy analysis (RDA) for the relationships between bacterial community composition and various environmental parameters at the phylum level in the mangrove sediments from Guangxi, China. Notes: T, temperature; OC, organic carbon; TN, total nitrogen; IP, inorganic phosphorus; OP, organic phosphorus; C/N, carbon/nitrogen ratio. * indicates *p* < 0.05; ** indicates *p* < 0.01.

**Figure 6:**
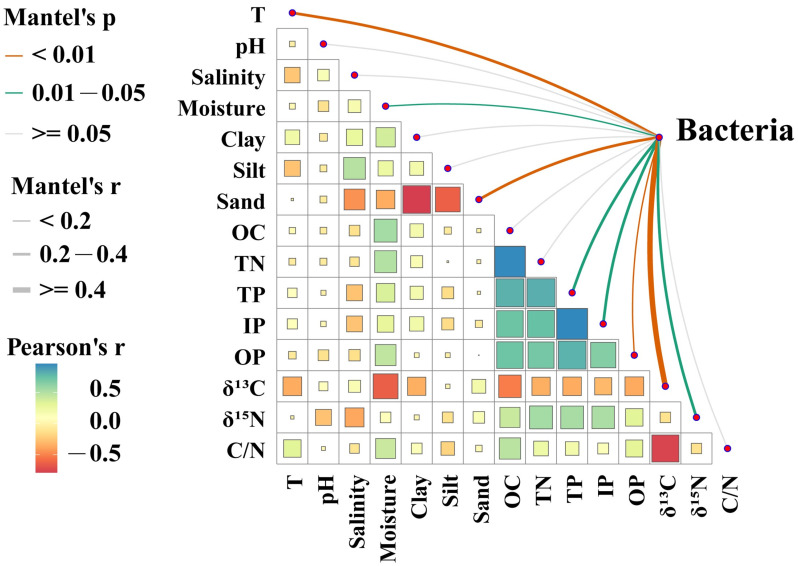
Mantel test showing the correlation between the sediment bacterial phyla and the physicochemical parameters in six mangrove habitats in Guangxi, China. The orange and green lines represent different significant correlation levels (*p* < 0.01 and *p* < 0.05, respectively), while the gray lines represent no correlation. The thickness of the lines corresponds to Spearman’s correlation coefficients, with thicker and thinner lines indicating stronger and weaker correlations, respectively. T, temperature; OC, organic carbon; TN, total nitrogen; TP, total phosphorus; IP, inorganic phosphorus; OP, organic phosphorus; C/N, carbon/nitrogen ratio.

**Figure 7:**
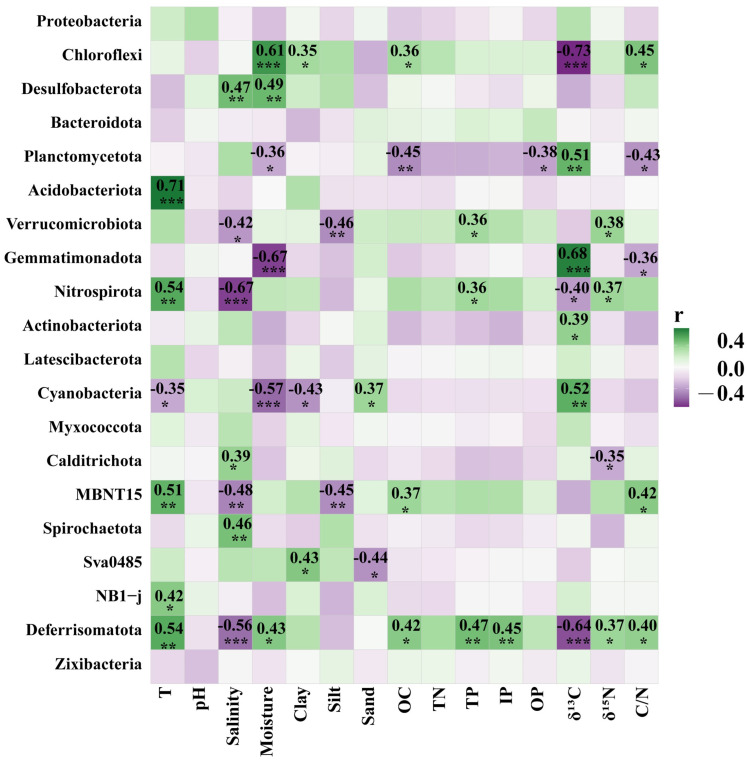
Correlation heatmap illustrating the relationship between the top 20 bacterial phyla and the sediment physicochemical properties among six different mangrove wetlands from Guangxi, China. Positive and negative correlations are indicated in green and purple, respectively. * indicates *p* < 0.05; ** indicates *p* < 0.01; *** indicates *p* < 0.001. Correlations between variables were calculated using the Spearman correlation coefficient. T, temperature; OC, organic carbon; TN, total nitrogen; TP, total phosphorus; IP, inorganic phosphorus; OP, organic phosphorus; C/N, carbon/nitrogen ratio.

**Figure 8:**
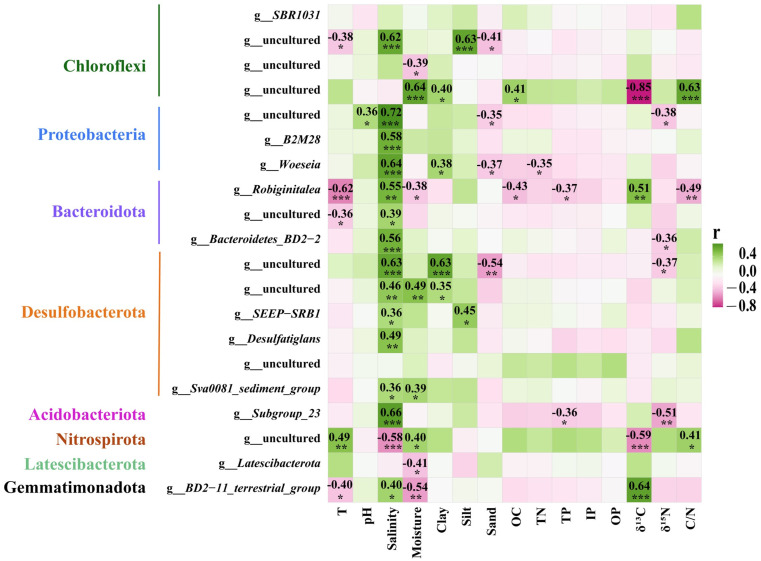
Correlation heatmap showing the relationship between the top 20 bacterial genera and the sediment physicochemical properties among six different mangrove wetlands from Guangxi, China. Positive and negative correlations are indicated in green and pink, respectively. * indicates *p* < 0.05; ** indicates *p* < 0.01; *** indicates *p* < 0.001. Correlations between variables were calculated using the Spearman correlation coefficient. T, temperature; OC, organic carbon; TN, total nitrogen; TP, total phosphorus; IP, inorganic phosphorus; OP, organic phosphorus; C/N, carbon/nitrogen ratio.

In order to determine which environmental factors affected the bacterial communities of the six mangrove sediments, the correlation between the bacterial communities and fifteen environmental variables (included temperature, pH, salinity, moisture, clay, silt, sand, OC, TN, TP, IP, OP, δ^13^C, δ^15^N, and C/N ratio were analyzed by redundancy analysis (RDA) and Mantel test (Figure 5, Figure 6, Figure 7 and Figure 8).

RDA indicated that bacterial communities responded differently to changes in the physicochemical properties of mangrove sediment at the phylum level. For instance, salinity, δ^13^C, temperature, δ^15^N, and silt were found to have significant correlations with the spatial distribution of mangrove bacterial communities (*p* < 0.05) (Figure 5). The selected environmental parameters can explain 24.01% of bacterial community variation in total. The contribution of the environmental factors to the bacterial community structure was in the following decreasing order: salinity (16.8%) > δ^13^C (15.5%) > temperature (12.1%) > δ^15^N (7.8%) > silt (7.4%) (Figure 5).

Spearman’s correlation further demonstrated that temperature, moisture, sand, TP, IP, OP, δ^13^C, and δ^15^N significantly affected the relative abundance of bacterial phyla (*p* < 0.05) (Figure 6).

As shown in Figure 7 and Figure 8, the two correlation heatmaps displayed the relationships between the bacterial community and the physicochemical parameters, exhibiting the significance of correlations between 15 different environmental variables and the top 20 bacterial phyla or genera. 

At the phylum level, Chloroflexi exhibited a significant negative correlation with δ^13^C, but it was positively correlated with moisture, clay, OC, and C/N ratio. Desulfobacterota was significantly positively affected by salinity and moisture. Planctomycetota displayed a negative correlation with moisture, OC, OP, and C/N, but a positive correlation with δ^13^C. Acidobacteriota was significantly positively correlated with temperature. Nitrospirota showed a negative correlation with salinity and δ^13^C but positive correlations with temperature, TP, and δ^15^N (Figure 7).

At the genus level, three uncultured Chloroflexi genera were negatively correlated with temperature, moisture, sand, and δ^13^C but were positively correlated with salinity, clay, OC, and C/N. *B2M28* displayed a positive correlation with salinity. *Woeseia* showed a positive correlation with salinity and clay but a negative correlation with sand and TN. *Robiginitalea* was significantly positively affected by salinity and δ^13^C but negatively affected by temperature, moisture, OC, TP, and C/N. *SEEP-SRB1*, *Desulfatiglans*, and the *Sva0081 sediment group* exhibited positive correlations with salinity (Figure 8).

### 3.6. Phylogenetic Analysis

**Figure 9:**
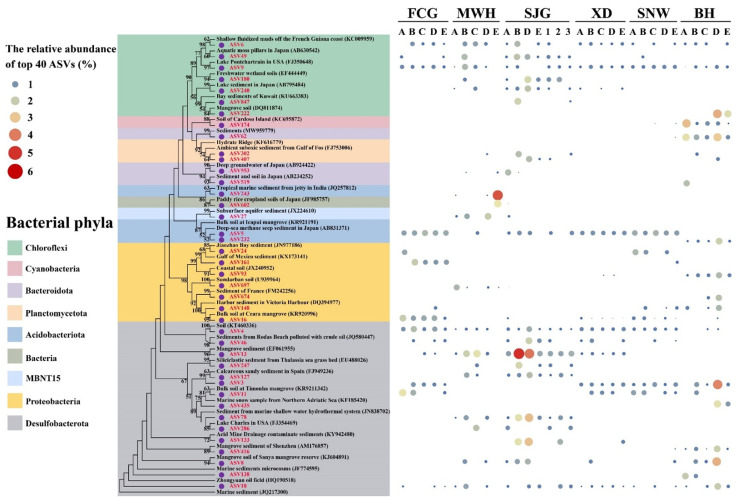
The phylogenetic tree of the top 40 most abundant ASVs observed in the six mangrove sites and their corresponding relative abundances are listed in the bubble chart at the right. The reference sequences in this tree represented all the sequences that share 94.9% or more nucleic acid identity in the NCBI database. Six scales of the bubble chart are shown at the left, and red bubbles and blue bubbles represent a relative abundance of ASV ≤ 6% and ≤1%, respectively. The ASVs that were affiliated with different phyla are highlighted with different colors.

To further reveal the taxonomic information of bacterial communities in mangrove sediments, the top 40 ASVs were selected to construct a phylogenetic tree, and the relative abundance of each ASV in six different sites was listed in the right bubble chart (Figure 9 and Table S1). Overall, the bacterial community in mangrove sediments exhibited a high diversity. The top 40 ASVs mainly belonged to eight phyla, namely Chloroflexi (7 ASVs), Cyanobacteria (1 ASVs), Bacteroidota (3 ASVs), Planctomycetota (2 ASVs), Acidobacteriota (3 ASVs), MBNT15 (1 ASVs), Proteobacteria (7 ASVs), and Desulfobacterota (15 ASVs) (Figure 9 and Table S1). 

In the Desulfobacterota phylum, the most abundant ASV13 was affiliated with the Desulfobacterota phylum, with the highest relative abundance reaching up to 5.0% at site SJG-2. The sequence of ASV13 had a 100% match with an uncultured delta proteobacterium clone XME70 (accession ID, EF061955) which originated from a mangrove sediment habitat. On the other hand, the ASV3 was also affiliated with the Desulfobacterota phylum, accounting for 3.9% of the highest value at site BH-D. The sequence of ASV3 had a 100% match with an uncultured delta proteobacterium clone OTU141 (JQ217300) which separated from the marine sediment (Figure 9 and Table S1).

Then, in the Chloroflexi phylum, the fourth most abundant ASV222 was a member of the Chloroflexi phylum and the Anaerolineae class, accounting for 3.3% of the highest value at site BH-D. The sequence of ASV222 had a 99.7% match with an uncultured Chloroflexi bacterium clone MSB-4H2 (DQ811874) from the mangrove soil. Also, in the Acidobacteriota phylum, the second most abundant ASV243 belonged to the Acidobacteriota phylum, accounting for 4.5% of the highest value at site MWH-E. The sequence of ASV243 had a 98.7% match with an uncultured bacterium clone KJ-UB-47 (JQ257812), which was obtained from the tropical marine sediment from the jetty in India. Furthermore, in the Cyanobacteria phylum, the fifth most abundant ASV174 was affiliated with the Cyanobacteria phylum and Synechococcales order, accounting for 3.1% of the highest value at site BH-A. The sequence of ASV174 had a 100% match with a *Synechococcus* sp. CENA180 (KC695872) from the soil of Cardoso Island (Figure 9 and Table S1). 

## 4. Discussion

Mangrove wetlands are important ecosystems and are distributed widely in tropical and subtropical tidal regions, providing protection, niches, and nutrients to all kinds of microbial communities [32]. Within mangrove ecosystems, microorganisms are the central players responsible for the biogeochemical cycling of nutrient elements and the process of energy flow in the mangrove sediments. Therefore, they are essential to the productivity, conservation, and rehabilitation of mangrove habitats [33,34]. In this study, we determined the bacterial diversity and communities in six stations (FCG, MWH, SJG, XH, SNW, and BH) of mangrove sediments in Guangxi Province, applying high-throughput sequencing technology. The influence of various physicochemical factors (such as temperature, pH, salinity, moisture, sediment grain size, OC, TN, TP, IP, OP, δ^13^C-org, δ^15^N, and C/N ratio) in shaping bacterial communities in the mangrove ecosystems was also investigated.

### 4.1. Distinct Bacterial Community Structures Shaping by Unique Mangrove Habitats

The patterns of spatial distribution in bacterial communities were significantly different among the six different mangrove sites from Guangxi Province, as demonstrated by NMDS, ANOSIM, and LDA (Figure 4). Different bacterial biomarkers appeared in different mangrove sediments, implying distinct bacterial community structures shaped by unique mangrove habitats. A number of previous studies also suggested that mangrove bacterial community structure was habitat-specific. The unique mangrove habitats at different geographic sites hosted bacteria with distinct community structures [6,35,36,37]. Possible reasons for this may be attributed to the wetland trophic status, salinity, organic carbon levels, and altering redox conditions [6,16,22,23,35].

Sediment microorganisms are important components of mangrove ecosystems and play essential roles in the biogeochemical cycling of nutrient elements, remineralization of organic matter, and degradation of contaminant [34]. In this study, Chloroflexi, Proteobacteria, Bacteroidota, and Desulfobacterota were identified as the most abundant bacterial communities in the surface sediments of six mangrove stations from Guangxi province (Figure 3a). A series of previous accumulating studies also demonstrated that these taxa were dominant bacterial phyla in the sediments of natural mangrove habitats [38,39,40,41]. For instance, Wu et al. (2016) showed that bacterial communities from mangrove rhizosphere sediments were mainly dominated by Proteobacteria, followed by Chloroflexi, Bacteroidetes, Planctomycetes, and Acidobacteria in Beilun Estuary National Nature Reserve, China [39]. Similarly, Zhang et al. (2018) investigated that the most abundant bacterial phyla mainly included Proteobacteria, Bacteroidetes, Actinobacteria, Acidobacteria, and Chloroflexi in the mangrove sediments of Daya Bay in the South China Sea [40]. Also, Ma and colleagues found that Proteobacteria, Chloroflexi, Acidobacteria, Planctomycetes, and Bacteroidetes were identified as the dominant top 5 bacterial phyla in the mangrove wetlands of Leizhou Peninsula, Zhanjiang, Guangdong province [38]. Furthermore, a related study revealed that the bacterial communities were composed of Proteobacteria, Bacteroidetes, and Firmicutes, with a high abundance of sulfate reducers, in the sediments of northern Red Sea mangroves along the coast of Saudi Arabia [41].

Most importantly, a series of accumulating evidences demonstrated that Chloroflexi had the genomic potential to degrade recalcitrant organic matter, such as lignin, halogenated compounds, and aromatic hydrocarbons in mangrove sediments [11,12,32,35,42]. In the present study, Chloroflexi was one of the most abundant bacterial phyla in the six mangrove sediment sites from Guangxi province, with the relative abundance reaching up to 31.6% at site SJG-2 (Figure 3a). In particular, the relative abundance of Chloroflexi was significantly positively affected by moisture, clay, OC, and C/N ratio but significantly negatively affected by δ^13^C (Figure 7). The *SBR1031* and several uncultured genera were the most abundant taxa in the Chloroflexi phylum, and they showed significant correlation with terrestrial-derived organic carbon (Figure 3b and Figure 8), which implied that many unknown Chloroflexi species exist in the mangrove wetlands, and their metabolic functions related to organic carbon degradation need to further study. Our findings may suggest that Chloroflexi plays an important role in the decomposition of organic carbon in mangrove ecosystems from Guangxi, China, agrees well with previous studies [32].

Desulfobacterota are known as the sulfate-reducing bacteria and are the main contributor to sulfur transformation in the mangrove ecosystem [3,34]. Desulfobacterota also play important roles in the cycling of C, N, and S from the mangrove intertidal zonation [19] and the anaerobic degradation of hydrocarbons in anoxic marine sediments [43]. In this study, Desulfobacterota was the fourth most abundant bacterial phyla in the six mangrove sediment sites from Guangxi province, with the relative abundance reaching up to 24.0% at site SJG-3 (Figure 3a). *Sva0081_sediment_group* and *SEEP-SRB1* were affiliated with the Desulfobacterota phylum, which was the predominant genera that appeared in the mangrove sites MWH-B and SNW-C, respectively, with a relative abundance ≥ 4.9% (Figure 3b). These results were consistent with previous studies which showed that Desulfobacterales were one of the dominant bacterial clusters in the mangrove wetland soil [3,34,44]. In the 32 mangrove sampling sites, Desulfobacterota displayed significantly positive correlations with salinity and moisture (Figure 7), and the genera *SEEP-SRB1*, *Desulfatiglans*, and *Sva0081 sediment group* in the Desulfobacterota phylum exhibited the most positive correlations with salinity (Figure 8). However, other studies observed that Desulfobacterales were significantly positively affected by OC and TN in a mangrove reserve located in the Merbok River estuary, Malaysia [3], which was different from our findings. Furthermore, many uncultured members of Desulfobacterota were also found at the mangrove stations FCG-C, SJG-2, and SJG-D (Figure 3b), which may imply unknown important bacterial taxa and the related ecological functions presented in the mangrove sediments from the six sites of Guangxi province.

Moreover, Proteobacteria, accounting for the highest proportion of bacteria, had been reported to play key roles in the biogeochemical cycling of carbon, nitrogen, and sulfur in the sediments of various mangrove ecosystems [10,45]. In our study, Proteobacteria were the second most abundant bacterial groups in the mangrove sediments from Guangxi province, with average values of 22.3 ± 4.5% in the whole samples and the highest values of 30.1% at the SNW-B site (Figure 3a). However, no significant correlation was observed between Proteobacteria and the 15 environmental parameters (Figure 7). The members of Bacteroidetes had been known to mediate the degradation of protein [10]. In our study, Bacteroidota were the third most dominant bacterial taxa in the surface sediments of mangrove sites, with the relative abundance over 24.6% at site BH-A (Figure 3a). However, no environmental variables significantly affected the relative abundances of Bacteroidota (Figure 7).

### 4.2. Effect of Various Environmental Factors on Bacterial Communities in Mangrove Sediments

Salinity has been recognized as one of the most important environmental parameters that drive the bacterial communities in sediments from different mangrove environments [3,21,35,46,47,48]. An increase in salinity is thought to have the largest effect on the physiochemical characteristics of freshwater sediments, and leads to dramatic changes from freshwater-adapted microbial species to facultative marine phytotypes [21]. A previous investigation demonstrated that salinity increased microbial decomposition rates and decreased the accumulation of soil organic matter in low-salinity wetlands [49]. Moreover, salinity could influence mineralization, immobilization, nitrification, and denitrification [48,50]. Our results suggested that salinity was the most important factor driving the spatial distribution of the bacterial communities in the six mangrove sediment sites, accounting for 16.8% of the contribution (*p* < 0.01) (Figure 5). A similar study reported that salinity was a key factor that governed the composition of bacterial communities in an intertidal mangrove sediment in Bhitarkanika, India [35]. A recent study also suggested that the biogeographic distribution of sediment bacterial communities in mangroves across China was driven mainly by salinity and the wetland trophic status [6]. In particular, the relative abundance of Desulfobacterota showed significant positive responses to salinity, whereas the relative abundance of Nitrospirota was significantly negatively affected by salinity in this work (Figure 7). Wang et al. (2018) found that salinity was a key factor driving the nitrogen cycling in the mangrove sediment and that nitrifying activity might be inhibited at higher salinity [51], which agreed well with our findings. A study revealed that the microbial community in a lake habitat with higher salinity exhibited the highest diversity and richness indices [52], which was consistent with our results.

Geochemical tracers such as the stable carbon isotope (δ^13^C), stable nitrogen isotope (δ^15^N), and elemental carbon/nitrogen (C/N) ratios are widely used to trace the sources and evaluate the fate of sediment organic matter (OM) in mangrove swamps [53]. Generally, sediment OM from terrestrial sources has lower δ^13^C and δ^15^N signatures and higher C/N ratios compared to OM derived from marine sources [53,54,55]. For instance, the C/N ratios of terrestrial vascular plants are > 12, the δ^13^C values of OM from terrestrial higher plants with a C_3_ pathway range from −33‰ to −24‰ (average, −27‰), and the typical δ^15^N values for terrestrial vascular plants range from −10‰ to +10‰ (average, ~2‰) [53,56]. In the present study, the values of δ^13^C, δ^15^N, and C/N ratios in the mangrove sediments ranged from −29.5‰ to −21.9‰, 0.8 to 8.7‰, and 11.5 to 22.6, respectively (Table 1). These results demonstrated that the sediment OM in the six stations the mangrove wetlands from Guangxi, China were mainly derived from terrestrial sources [53]. Meanwhile, δ^13^C was significantly negatively correlated with the spatial distribution of bacterial communities in the mangrove sediments of six sites, but they were significantly positively affected by δ^15^N (Figure 6). These findings were in agreement with a previous study that indicated that δ^15^N and δ^13^C possibly influenced the bacterial community structure in sediments from the Xisha Trough, South China Sea [57]. Specifically, δ^13^C showed significant negative effects on the relative abundances of Chloroflexi and Nitrospirota but significant positive influences on the relative abundances of Cyanobacteria and Planctomycetota in the mangrove sediments (Figure 7). It is well known that mangrove sediments contain more lignin and that lignin acts as an indicator of the terrestrial-derived OC; therefore, the organic matter of mangrove sediments mainly originated from a terrestrial source [11]. Several studies suggested that Chloroflexi (*Dehalococcoidales*) could be involved in the degradation of more recalcitrant aromatic compounds (such as lignin) in mangrove sediments [10,42,58]. These findings were in agreement with our studies. However, another investigation reported that δ^13^C and Chloroflexi were positively correlated, while δ^13^C and Planctomycetes were negatively correlated from the core sediments of the South China Sea [57]. One possible reason may be that the OM of sediments in the South China Sea may be largely derived from marine [57]. Moreover, δ^15^N was significantly positively correlated to Nitrospirota. The C/N ratio significantly positively affected Chloroflexi but significantly negatively affected Planctomycetota (Figure 7). Wang et al. (2018) also demonstrated that the composition of the bacterial community was mainly explained by OC-related variables, including stable carbon isotopes (δ^13^C) and carbon to nitrogen ratio (C/N) in sediments of two China marginal seas [59]. Also, sediments with microbial mats contained higher OC/TN ratios and lower δ^13^C values in a marine mangrove [60]. These findings agreed well with our study. 

Temperature was found to be an important driver of the seasonal patterns of bacterial communities in sediments of subtropical coastal wetland [61] and tidal flats from the Yellow River Delta [62]. Temperature also affected the growth rates and respiration rates of microbial communities, mainly by altering the rate of enzyme activity and the efficiency of membrane transport [63,64]. In our study, the temperature of the six mangrove surface sediments from Guangxi Province ranged from 24.2 to 30.7 °C; the highest and lowest values were found at sites MWH-D and BH-C, respectively (Table 1). Temperature had significant correlations with the spatial distribution of mangrove bacterial communities, and the relative abundances of Acidobacteriota and Nitrospirota were significantly positively affected by temperature, but the relative abundance of Cyanobacteria was significantly negatively affected by temperature (Figure 7). Our results were in agreement with studies on other wetlands [61] and hot springs [65]. For instance, the richness of phototrophic Cyanobacteria decreased with increasing temperature in hot springs from Yellowstone National Park [65]. 

Moreover, soil texture and mineralogy are reported to be important drivers of microbial communities in many studies [66,67,68,69]. Given that soil is a mixture of minerals, most soil microorganisms live attached to the surfaces; thus, distinct bacterial communities were selected [68]. Soil particle size significantly influenced microbial community structure, with higher microbial diversity occurring in small soil particles than in large [66,67]. The lower diversity of microbial communities in larger soil particles could be attributed to several factors, including low nutrient availability, protozoan grazing, and competition with fungal organisms [66]. Jackson and Weeks (2008) also demonstrated that particle size might be more important in shaping bacterial communities than nutrient status [67]. Furthermore, Deng et al. (2010) revealed that moisture could control soil microbial communities and respiration rates in subtropical forest ecosystems [70]. In this work, the textures of surface mangrove sediments were mainly characterized by clayey silt and sandy silt (Table 1). Also, sediment grain sizes such as silt had a significant influence on the distribution of bacterial communities in the six stations of mangrove sediments (*p* < 0.05) (Figure 5). Clay significantly positively affected Chloroflexi but negatively affected Cyanobacteria. Sand had significantly positive effects on Cyanobacteria (Figure 7). This result was consistent with what was observed in a previous study, which affirmed the silt–clay percentages as being the most important driver of both bacterial and archaeal communities in the Pacoti River mangrove sediments of the northeast Brazil [69]. 

The pH values of mangrove soils changed depending on various factors such as geographical region, season, aridity, mangrove species, and pollution. The pH values of mangrove sediments affected the bacterial community structure. A decrease in pH resulted in a higher number of acidophiles and a lower number of mesophiles and alkalophiles [37,69]. In the present study, the pH values of sediments ranged from 5.8 to 9.0, suggesting that the mangrove sediments from Guangxi province ranged from acidic to alkaline (Figure 2b). However, pH did not affect the bacterial community in the sediments of six mangrove stations. Similarly, OC, TN, TP, IP, and OP also had no effects on the composition of bacterial community in the mangrove sediments. 

## 5. Conclusions

This is the first comprehensive investigation of the composition and spatial variability in sediment bacterial communities, along with key environmental driving factors, across six representative mangrove wetlands in Guangxi, China. Notably, significant variations in the diversity and structure of sediment bacterial communities were observed among the different geographic locations of the mangrove wetlands. The bacterial communities were predominantly comprised of Chloroflexi, Proteobacteria, Bacteroidota, and Desulfobacterota. Furthermore, the key environmental variables, including salinity, δ^13^C, temperature, δ^15^N, and silt content, appeared to play crucial roles in determining the spatial distribution of sediment bacterial communities within the mangrove ecosystems. Specifically, Chloroflexi may play a pivotal role in the degradation of terrestrial-derived organic carbon. Our findings contribute to a deeper understanding of how bacterial communities respond to various environmental factors across different geographic sites within mangrove ecosystems.

## Figures and Tables

**Figure 1:**
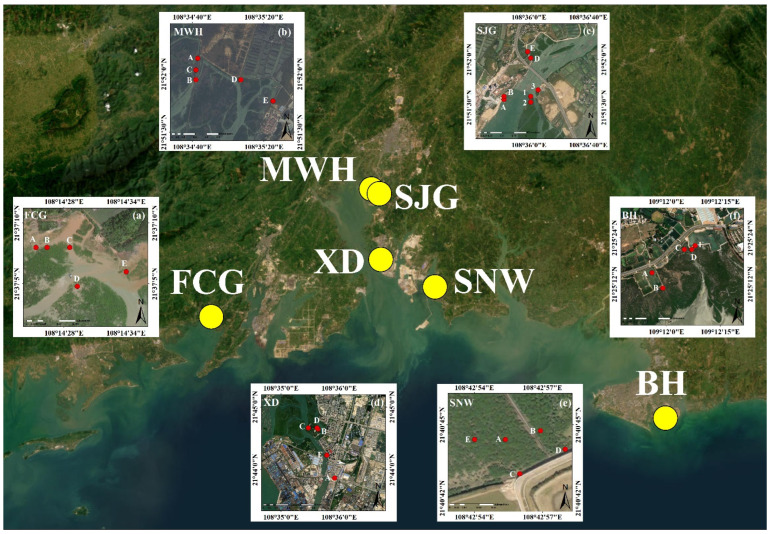
The six sampling sites in the mangrove wetlands from Guangxi, China. (**a**) FCG refers to Fangcheng Port Mangrove Natural Reserve (sites A, B, C, D, and E), (**b**) MWH refers to Maowei Sea Mangrove Natural Reserve (A, B, C, D, and E), (**c**) SJG refers to Shajing Port mangrove wetland (A, B, D, E, 1, 2, and 3), (**d**) XD refers to Xiandao Park mangrove wetland (A, B, C, D, and E), (**e**) SNW refers to Sanniang Bay mangrove wetland (A, B, C, D, and E), and (**f**) BH refers to Bei Sea Mangrove Natural Reserve (A, B, C, D, and E).

**Table 1:** Physicochemical parameters of the surface sediment samples from the six mangrove sites from Guangxi, China.

Stations	Longitude (E)	Latitude (N)	Temperature (°C)	pH	Salinity (psu)	Water Content (%)	Clay (0–4 μm)	Silt (4–64 μm)	Sand (64–2000 μm)	OC (%)	TN (%)	TP (μmol/g)	IP (μmol/g)	OP (μmol/g)	δ^13^C (‰)	δ^15^N (‰)	C/N
FCG	108.24	21.62	27.4 ± 0.4	7.5 ± 0.7	5.5 ± 1.8	37.9 ± 6.8	22.6 ± 10.2	61.7 ± 5.1	15.7 ± 12.5	1.3 ± 1.1	0.1 ± 0.1	10.9 ± 5.2	7.2 ± 2.7	3.7 ± 2.5	−26.3 ± 0.4	4.5 ± 0.7	17.5 ± 1.6
MWH	108.58	21.87	28.4 ± 1.5	6.9 ± 0.7	1.4 ± 1.0	41.9 ± 17.1	16.9 ± 4.5	56.4 ± 10.7	26.7 ± 11.2	2.2 ± 1.3	0.2 ± 0.1	16.1 ± 8.6	10.5 ± 7.5	5.6 ± 3.6	−25.5 ± 2.2	6.1 ± 2.4	16.0 ± 2.8
SJG	108.60	21.86	28.0 ± 0.8	7.3 ± 0.1	1.5 ± 0.5	46.0 ± 12.4	23.3 ± 12.1	61.0 ± 9.1	15.7 ± 11.8	2.2 ± 1.7	0.1 ± 0.1	19.0 ± 7.6	13.2 ± 6.4	5.8 ± 2.4	−27.9 ± 1.0	5.5 ± 1.6	17.7 ± 2.8
XD	108.60	21.73	29.5 ± 1.1	7.3 ± 0.8	3.0 ± 0.6	37.0 ± 9.2	26.7 ± 13.9	58.1 ± 8.1	15.2 ± 13.1	1.9 ± 0.8	0.1 ± 0.0	17.3 ± 3.1	11.0 ± 2.1	6.3 ± 1.5	−27.1 ± 0.4	3.6 ± 1.7	19.4 ± 2.4
SNW	108.72	21.68	26.7 ± 1.0	7.1 ± 0.8	5.7 ± 1.5	41.7 ± 16.2	21.1 ± 3.2	71.3 ± 4.4	7.6 ± 6.4	0.7 ± 0.6	0.1 ± 0.0	8.6 ± 3.2	5.7 ± 2.3	2.9 ± 1.1	−25.1 ± 1.2	4.4 ± 1.5	13.2 ± 1.1
BH	109.20	21.42	24.6 ± 0.4	7.4 ± 1.1	3.0 ± 1.4	27.0 ± 5.9	13.0 ± 2.2	62.0 ± 12.0	25.1 ± 13.9	1.4 ± 0.8	0.1 ± 0.1	16.4 ± 11.9	10.8 ± 8.7	5.6 ± 3.6	−23.6 ± 1.2	4.7 ± 1.3	13.8 ± 1.6

## Data Availability

All raw sequence datasets of 16S rRNA genes from this study have been deposited into the NCBI Sequence Read Achieve (SRA) database with accession no. PRJNA1177219.

## Supplementary Materials

The following supporting information can be downloaded at: https://www.mdpi.com/article/10.3390/microorganisms12122607/s1, Table S1: The taxanomy classification, NCBI reference sequences, isolation source, percent identity, and accession ID of the top 40 ASVs in the surface sediment samples from the six mangrove sites of Guangxi, China.
